# Effects of Different Formulations of Exogenous Nutrient Bag Waste on Soil Properties and Physiological Activities and Root Transcriptomic Responses of Oat

**DOI:** 10.3390/biology15201794

**Published:** 2026-10-09

**Authors:** Li Gong, Le Wang, Liping Su, Wei Sa, Quanmin Dong

**Affiliations:** 1 Research Institute of Science and Technology, Qinghai University, Xining 810016, China; gongli@qhu.edu.cn (L.G.);; 2 Key Laboratory of Adaptive Management for Alpine Grassland Ecosystem of Qinghai Province, Qinghai University, Xining 810016, China

**Keywords:** oat, exogenous nutrient bag, transcriptome, WGCNA

## Abstract

This study used typical chestnut soil from Huangyuan County, Qinghai Province as the experimental soil, and selected three oat varieties, “Baiyan 7”, “Qingyan 2”, and “Qinghai 444”, for mixed sowing. A blank control (CK) and five treatments with different ratios of exogenous nutrient bag waste (PF-R-1 to PF-R-5) were established. Through soil physicochemical analysis, microbial biomass determination, RNA sequencing (RNA-Seq), and weighted gene co-expression network analysis (WGCNA), the effects of exogenous nutrient bag waste with different ratios soil nutrient availability, microbial activity and root gene expression in oats were systematically evaluated. The results showed that most treatments enhanced soil organic matter, nitrogen content, and microbial activity. Transcriptomic analysis revealed that the comparison between PF-R-3 and PF-R-2 treatments exhibited the highest total number of differentially expressed genes (DEGs), with enrichment in pathways related to ribosomal protein translation, oxidative phosphorylation energy metabolism, and DNA damage repair. These enrichment patterns suggest potential changes in energy metabolism and protein synthesis, although direct functional validation was not performed. Moreover, DEGs uniquely identified in PF-R-3 were significantly enriched in nitrogen metabolism and secondary metabolite biosynthesis pathways; WGCNA identified the coral2 module as the core regulatory module, with its genes predominantly enriched in functions related to transmembrane transport and plant hormone signal transduction, which were highly consistent with efficient root nutrient acquisition and the maintenance of metabolic homeostasis. Fluorescence staining confirmed the successful colonization of oat root tissues by Morchella mycelium. Taken together, based on the integration of soil nutrient supply stability, root gene expression regulation, and fungal colonization evidence, the PF-R-3 treatment (wheat + corncob + oat) emerged as a promising candidate among the tested formulations under the experimental conditions. However, further field trials incorporating yield and quality data are needed before a definitive recommendation can be made. This study provides a theoretical basis and technical support for the resource utilization of exogenous nutrient bag waste and the green cultivation of oat in alpine regions.

## Simple Summary

The extensive accumulation of agricultural waste (such as wheat straw and corncob) poses environmental challenges in alpine regions. Returning these wastes to the field after fermentation, known as ENB waste, can improve soil conditions, but the optimal combination of different waste materials for oat cultivation remains unclear. In this study, we tested several mixed substrates containing ENB waste in oat fields and examined how they affect soil nutrients, oat root gene expression, and fungal associations. We found that a mixture of wheat straw, corncob, and oat residues (PF-R-3) maintained relatively stable soil nutrient levels throughout the growing season. At the molecular level, this treatment triggered stronger gene expression changes in oat roots related to energy production and nutrient uptake compared with other mixtures. We also observed that beneficial Morchella fungi were present on oat roots, which might contribute to these positive effects. Future trials are needed to confirm its agronomic performance before farmers can adopt this method.

## 1. Introduction

Oat (*Avena sativa* L.) is an important dual-purpose crop for both grain and forage in the alpine agro-pastoral ecotone, characterized by cold tolerance, tolerance to infertile soils, and broad adaptability, and it occupies a vital position in agricultural production in Qinghai Province and across the Qinghai–Tibet Plateau [1]. However, this region is constrained by natural conditions such as high altitude, low temperature, and short growing season, resulting in slow soil organic matter mineralization rates and prolonged nutrient turnover cycles. Furthermore, coupled with the expansion of continuous oat cropping area and increasing dependence on chemical fertilizers in recent years, problems such as soil degradation and declining soil fertility have become increasingly severe, and have become a key bottleneck restricting the green development of the oat industry [2]. Field experiments conducted by Fu Dongqing [3] in the Shihezi region of Xinjiang showed that the ‘Qinghai 444’ oat achieved the highest hay yield of 15,310 kg hm^−2^ under a planting density of 312 kg hm^−2^ and a nitrogen application rate of 160 kg hm^−2^. This high yield potential, however, is highly dependent on adequate soil nutrient supply. Under the soil degradation conditions described above, such yield levels are difficult to sustain without external organic amendments.

*Morchella* spp. are a group of rare edible and medicinal fungi. Their artificial cultivation requires the addition of exogenous nutrient bags (ENB) to the soil to supplement carbon and nitrogen sources and optimize the mycelial growth substrate [4]. During Morchella cultivation, the application rate of exogenous nutrient bags ranges from 2 to 4 bags per square meter. Studies have shown that, within a certain range, the application amount of exogenous nutrient bags is positively correlated with the fruiting yield of Morchella [5,6,7]. Therefore, after cultivation, large quantities of ENB waste (rich in incompletely decomposed agricultural and forestry by-products such as wheat, corncobs, alfalfa, and oats) are often directly discarded or incinerated, resulting in both resource waste and agricultural pollution. It has been demonstrated that ENB waste is rich in various nutritional components essential for the growth and reproduction of edible fungi, including crude protein, crude fat, crude fiber, polysaccharides, trace elements, vitamins, and minerals [8]. Notably, the residual mycelium and its metabolites from Morchella cultivation may carry specific bioactive substances. When returned to the field along with the organic materials, these residues may influence root development, nutrient uptake, and stress tolerance of subsequent crops. However, systematic research on the direct use of ENB waste as a soil amendment for subsequent oat cultivation remains lacking. The nutrient release dynamics under different ratios, their regulatory effects on the soil microecological environment, and the molecular regulatory mechanisms underlying oat root growth and development remain unclear.

The addition of organic materials can alter the soil carbon-to-nitrogen ratio, enzyme activities, and micro-environmental conditions, thereby reshaping the structure and function of soil microbial communities, and ultimately affecting key physiological processes in crop roots, such as nutrient uptake, hormone signal transduction, and stress responses [9,10]. Previous studies have demonstrated that the return of lignocellulose-rich organic wastes to the field can significantly increase soil organic matter content and microbial biomass carbon and nitrogen, while upregulating the expression of genes related to nitrogen transport, antioxidant defense, and auxin signaling in crop roots [11,12]. In recent years, high-throughput RNA sequencing (RNA-Seq) and weighted gene co-expression network analysis (WGCNA) have been widely applied in research on crop–microbe interactions, enabling the systematic elucidation of the molecular regulatory networks underlying root responses to external environmental changes.

In this study, typical chestnut soil from Huangyuan County, Qinghai Province was used as the experimental soil, and three local oat varieties, “Baiyan 7”, “Qingyan 2”, and “Qinghai 444”, were selected for mixed sowing. A blank control (CK) and five treatments with different ratios of ENB waste (PF-R-1 to PF-R-5) were established. This study was designed to address the following three aspects: (1) dynamically monitoring the changes in soil physicochemical properties, enzyme activities, and microbial biomass carbon, nitrogen, and phosphorus under different treatments to elucidate soil nutrient availability and microbial activity; (2) analyzing differentially expressed genes (DEGs) in the oat root transcriptome using RNA-Seq technology, and identifying key metabolic pathways through GO and KEGG enrichment analysis; (3) constructing co-expression networks via WGCNA to identify core regulatory modules closely associated with nutrient supply, and verifying the colonization of Morchella mycelium in oat roots using fluorescence staining techniques. By integrating multidimensional data from soil nutrient availability, microbial activity, plant root gene expression, and mycelium–root associations, this study aimed to systematically evaluate the ameliorative effects of returning ENB waste to the field, elucidate the molecular response mechanisms underlying mycelium–root interactions, and screen the optimal treatment ratio, thereby providing a theoretical basis and technical support for the resource utilization of Morchella waste and the green cultivation of oat in alpine regions.

## 2. Materials and Methods

### 2.1. Description of the Experimental Site

The experimental site was located at a farmer’s field in Sitan Village, Riyue Township, Huangyuan County, Xining City, Qinghai Province (36°31′53″ N, 101°7′24″ E). The area has a plateau continental climate with predominantly chestnut soil.

### 2.2. Experimental Materials

The oat varieties used in this study were “Baiyan 7”, “Qingyan 2”, and “Qinghai 444”, all of which are locally adapted oat varieties in Qinghai. For each variety, 7 kg of seeds were provided and sown as a mixture. The total formulation is given as component percentages in Table 1, and the actual application rate was 29 kg of exogenous nutrient bag waste per plot (25 m^2^), equivalent to 1.2 kg m^−2^. The remaining 10% of the ENB waste is quicklime.

### 2.3. Experimental Design

The experiment was laid out in a randomized block design with a total of six treatments: blank control (CK); addition of wheat and corncob (PF-R-1); addition of wheat, corncob, and alfalfa (PF-R-2); addition of wheat, corncob, and oat (PF-R-3); addition of wheat and alfalfa (PF-R-4); and addition of wheat and oat (PF-R-5). The exogenous nutrient bags, consisting of spent waste from Morchella cultivation, were collected at the end of March 2025. The experiment consisted of six treatments (a three-variety mixture of oats at a 1:1:1 ratio by seed number) with three replicates, with 1 m spacing between plots. Oats were drill-sown on 8 May 2025, at a rate of 17 kg ha^−1^, and harvested on 14 October 2025. Field management followed standard local practices. The experiment was arranged in a randomized complete block design (RCBD) with six treatments and three replications, giving a total of 18 experimental units (plots). Each treatment was randomly assigned to one plot within each of the three blocks, so that every block contained all six treatments. Each plot had an area of 25 m^2^, and the total plot area was 18 × 25 m^2^ = 450 m^2^, excluding border areas.

### 2.4. Sample Collection

Oat root samples were collected on 28 July 2025. The roots were placed in sterile 10 mL centrifuge tubes, with three replicates per treatment, stored in a foam box with dry ice, and immediately sent to Guangzhou Gidio Biotechnology Co., Ltd (Guangzhou, China). for root transcriptome sequencing. Soil samples were collected on 8 May, 28 July, and 14 October 2025. After soil was sampled from the 0–20 cm layer after removing surface litter, soil samples were taken with three replicates per treatment. The soil samples were air-dried indoors, ground, and passed through a sieve for the determination of soil physicochemical properties.

Data were analyzed using a two-way repeated-measures ANOVA (SPSS Statistics 24) with treatment and sampling time as fixed factors and block as a random factor. Tukey’s HSD test was used for multiple comparisons at *p* < 0.05. Root samples were collected from 6 randomly selected points within each plot, with 3 plants sampled per point. Root tissues were pooled and mixed thoroughly before RNA extraction to obtain a composite sample representing the average transcriptional profile of the mixed stand.

### 2.5. Determination of Indicators

We quantified the key soil chemical properties using established procedures: soil organic matter was determined by the potassium dichromate heating method [13], and Total nitrogen was measured using a flow analyzer (model Auto Analyzer 3-AA3, manufactured by SEAL, Norderstedt, Germany) after digestion with sulfuric acid and an accelerator [14]. Alkali-hydrolyzable nitrogen was measured via the alkali diffusion method [15]. Available potassium was extracted with ammonium acetate and determined by flame atomic absorption spectrometry (PinAAciie 900F, PerkinElmer, Waltham, MA, USA) [16]. Microbial biomass carbon (MBC) was analyzed with a TOC analyzer (TOC-L, Shimadzu, Kyoto, Japan) [17]. Microbial biomass nitrogen (MBN) and phosphorus (MBP) were determined by chloroform fumigation followed by flow injection analysis [18]. Enzyme activities were assessed as follows: urease by indophenol blue colorimetry [19], protease activity was determined by a spectrophotometric method, and catalase activity via potassium permanganate titration [20].

We analyzed oat root data on the Gidio (Guangzhou, China) cloud platform (https://www.omicsmart.com) (accessed on 20 September 2026).

### 2.6. Transcriptome Library Construction and Sequencing Workflow

In this study, different combinations of wheat, corncob, alfalfa, oat, and other materials were used as experimental treatments. After oat root samples were collected, immediately snap-frozen in liquid nitrogen, and then stored at −80 °C for subsequent RNA extraction, library construction, and transcriptome sequencing.

After total RNA was extracted from oat roots, rRNA was removed using a standard kit to enrich mRNA. The resulting mRNA was selectively enriched by mRNA Capture Beads. Following magnetic bead purification, the mRNA was fragmented at high temperature. Using the fragmented mRNA as a template, first-strand cDNA was synthesized in a reverse transcription mixture, followed simultaneously by second-strand cDNA synthesis, end repair, and A-tailing. Adapters were then ligated, and after purification, target fragments were selected. The library was subsequently amplified by PCR. After passing quality inspection, the sequencing libraries were subjected to high-throughput sequencing. The transcriptome sequencing was commissioned to Guangzhou Gidio Biotechnology Co., Ltd.

### 2.7. Sequencing Data Filtering, Assembly, and Expression Quantification

Raw sequencing reads were first quality-filtered using fastp (v0.18.0) to remove low-quality sequences (bases with Q ≤ 20 accounting for more than 50% of a read), sequencing adapters, reads consisting entirely of A bases, and reads with N content exceeding 10%. The resulting clean reads were used for downstream analysis. Sequencing was performed on the Illumina NovaSeq X Plus platform (Illumina, San Diego, CA, USA) with 150 bp paired-end reads, generating approximately 6.35 Gb of data per sample. The Q20 and Q30 values were above 98% and 94%, respectively. Clean reads were aligned to the Avena sativa cv. ‘Sang’ reference genome (PepsiCo_Oat_OT3098_V2; https://wheat.pw.usda.gov/GG3/graingenes-downloads/pepsico-oat-ot3098-v2-files-2021) (accessed on 20 September 2026) using HISAT2 (v2.1.0), with a mapping rate ranging from 63.8% to 86.8%, with an average of 71.9%.

### 2.8. Sample Relationship Analysis

Based on the calculated gene expression levels, principal component analysis (PCA) and correlation analysis (based on Pearson correlation coefficients) were conducted to evaluate the clustering patterns and correlations among samples.

### 2.9. Differential Expression Analysis

Differentially expressed genes (DEGs) were identified using DESeq2 (v1.24), with the thresholds set at a false discovery rate (FDR) < 0.05 and |log_2_ fold change| > 1.

### 2.10. GO and KEGG Annotation and Enrichment Analysis of Differentially Expressed Genes

Significantly enriched Gene Ontology (GO) terms were identified using the hypergeometric distribution test, and the distribution of differentially expressed genes (DEGs) within each functional category was statistically analyzed. Pathway enrichment analysis was conducted based on the KEGG database, and the same statistical method was applied to screen for key metabolic pathways and regulatory networks (KEGG database website: https://www.kegg.jp/kegg/docs/relnote.html (accessed on 11 September 2026). GO database website: https://www.geneontology.org/docs/ontology-documentation/) (accessed on 11 September 2026).

### 2.11. Weighted Gene Co-Expression Network Analysis

The weighted gene co-expression network was constructed using the WGCNA package in R software (version 4.1.2), based on the filtered TPM values and mycelial physiological indicator data. First, hierarchical clustering analysis was performed on the 18 samples using the hclust function to identify and remove outlier samples. Using the retained samples, the pickSoftThreshold function was employed to calculate pairwise Pearson correlation coefficients between genes to construct an adjacency matrix. The optimal soft-thresholding power was determined when the scale-free topology fitting index (R^2^) reached 0.85. The adjacency matrix was then transformed into a topological overlap matrix (TOM) using the adjacency function. Based on the TOM, a weighted gene co-expression network was constructed, and genes with similar expression patterns were clustered into the same modules, each assigned a specific color. After module construction, the module eigengene (ME) was calculated, and Pearson correlation coefficients(r) and corresponding *p*-values between the ME and two traits—straw addition amount (kg/plot) and mycelial growth rate (relative Morchella colonization rate)—were computed. Modules with |r| ≥ 0.35 and *p* < 0.01 in at least one trait were selected as target modules.

### 2.12. WGA-PI Staining of Fungal Mycelia in Oat Roots

First, prepare a 0.2% Tween-PBS solution by dissolving 0.2 mL of Tween-20 in 100 mL of PBS. Then, mix 970 μL of this solution with 10 μL of WGA-AF488 stock solution and 20 μL of propidium iodide (PI) stock solution. The staining solution should be prepared freshly before use and stored protected from light. Subsequently, the roots were fixed with Carnoy’s fixative to remove chlorophyll. The roots were then transferred into 10% KOH solution, sealed, and incubated at 85 °C for clearing. After clearing, the tissues were washed 4–5 times with PBS for pre-staining treatment; The prepared WGA-PI staining solution was added to the centrifuge tubes containing the roots, and vacuum infiltration was performed using a vacuum pump for 4 cycles, each lasting 5 min, with a 5 min interval at atmospheric pressure between cycles. After staining, the roots were washed 2–3 times with PBS, mounted with an anti-fade mounting medium, and stored at 4 °C in the dark. Finally, images were captured using a fluorescence microscope or confocal microscope. The staining of the roots was commissioned to Servicebio Biotechnology Co., Ltd. (Wuhan, China).

## 3. Results

### 3.1. Effects of Exogenous Nutrient Bag Waste on Soil Properties and Physiological Activities

The dynamic changes in soil physicochemical properties (Table 2) and soil biological properties (Table 3) under different ENB waste treatments were examined at three stages: before oat sowing, the mid-growth stage (81 d), and the harvest stage (159 d). The results revealed significant differences in the ameliorative effects on soil among treatments, with distinct patterns of nutrient release and activity changes emerging as the growth period progressed. The changes in total nitrogen were relatively stable, mostly ranging between 1.5 and 2.8 g kg^−1^. At 159 days, the PF-5 treatment group reached 2.8 g kg^−1^, significantly higher than the other groups. Urease showed an overall downward trend, decreasing from around 200–300 μg g h^−1^ before sowing to 170–210 μg g h^−1^ at 159 days. Microbial biomass carbon was relatively high before sowing (456–751 mg kg^−1^), peaked at 81 days (approximately 600–950 mg kg^−1^), indicating vigorous microbial reproduction at that time, but dropped sharply to 189–449 mg kg^−1^ at 159 days. The soil organic matter, nitrogen content, and microbial biomass carbon, nitrogen, and phosphorus levels in all treatments were increased. The PF-R-1 treatment exhibited the highest levels of soil organic matter, total nitrogen, alkali-hydrolyzable nitrogen, and the activities of urease, protease, and microbial biomass carbon, nitrogen, and phosphorus, whereas the PF-R-5 treatment showed significantly lower basal nutrient contents and biological activities. Meanwhile, PF-R-2 exhibited a relatively high initial electrical conductivity (EC) value, posing a potential risk of short-term salt stress, while the pH in the CK treatment was significantly higher than that in all ENB treatments, reflecting the early-stage regulatory effect of ENB waste on the soil saline–alkali environment. At the mid-growth stage of oat (81 d), the effects of ENB waste on MBC were treatment-specific. MBC in PF-R-1 and PF-R-2 decreased slightly from pre-sowing levels, while it increased in PF-R-3, PF-R-4, and PF-R-5, with PF-R-4 showing the highest value, while electrical conductivity (EC) values decreased significantly, markedly alleviating the risk of salt stress. Notably, soil nutrient levels and microbial activity in the PF-R-4 treatment surged to a peak, demonstrating strong mid-term nutrient supply capacity, whereas the PF-R-3 treatment maintained moderate to high nutrient supply and stable microbial activity. At the harvest stage (159 d), nutrient release tended to stabilize. The PF-R-5 treatment released large amounts of carbon and nitrogen nutrients at the late stage, and soil enzyme activities increased significantly, whereas the nutrients in the PF-R-1 treatment had been largely consumed during the early stage, resulting in a marked decline in soil microbial activity. The PF-R-3 treatment still maintained moderate to high levels of soil nutrient content and biological activity, with a balanced and stable nutrient supply pattern, while the PF-R-4 treatment consistently maintained relatively high levels of soil nutrients and biological activity. Overall, the ENB waste treatments effectively increased soil organic matter, nitrogen content, and microbial activity, improved soil biological properties, and exerted a certain regulatory effect on soil pH. The PF-R-3 treatment, characterized by balanced and stable nutrient supply, consistently maintained soil nutrient content and biological activity at medium to high levels throughout the entire growth period, while simultaneously providing saline–alkali amelioration effects.

### 3.2. Effects of Different Treatments on Oat Root Gene Expression

**Figure 1:**
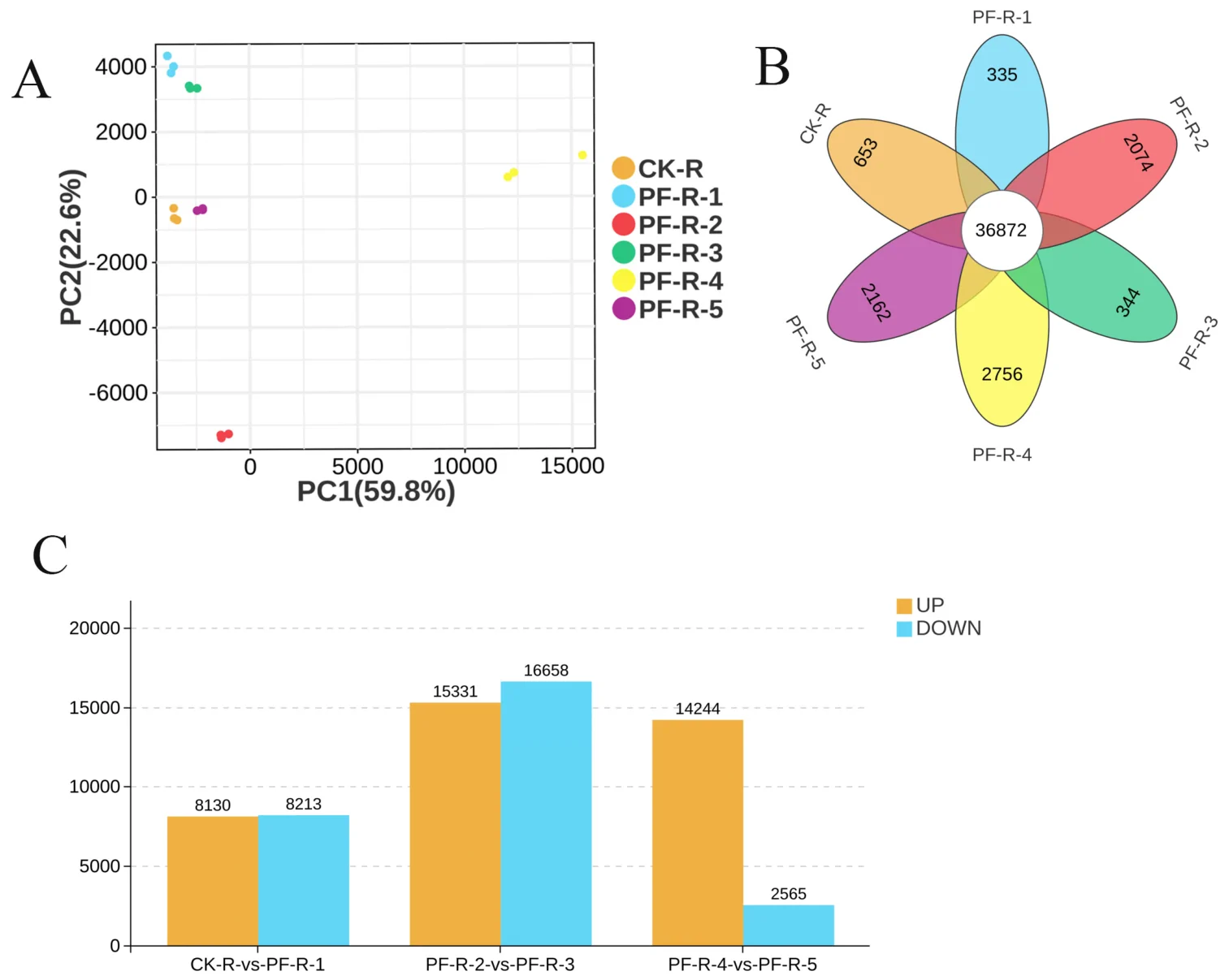
Analysis of RNA-Seq results: (**A**) Principal component analysis; (**B**) Venn diagram; (**C**) Statistics of differentially expressed genes in each group.

RNA-Seq was performed on the six sample groups (CK, PF-R-1, PF-R-2, PF-R-3, PF-R-4, and PF-R-5). A total of 36,872 core sequences were shared among all groups, constituting the major component of the transcriptome. The number of unique sequences varied markedly among groups. PF-R-4 exhibited the highest number of unique sequences (2756), followed by PF-R-5 (2162) and PF-R-2 (2074), while CK (653), PF-R-1(335), and PF-R-3(344) had considerably fewer. PCA results showed that the first principal component (PC1) explained 59.8% of the total variance, and the second principal component (PC2) explained 22.6%, yielding a cumulative contribution rate of 82.4%, which adequately represented the overall gene expression differences among samples and could be used for subsequent analysis (Figure 1A). Differential expression analysis was performed among different treatment groups. Between CK and PF-R-1, the numbers of up- and down-regulated sequences were approximately equal, indicating a bidirectional and balanced expression perturbation induced by the treatment. The comparison between PF-R-2 and PF-R-3 exhibited the highest total number of differential sequences, with the number of down-regulated sequences slightly exceeding that of up-regulated sequences, suggesting the most pronounced transcriptional divergence between the two treatments. It should be noted that this transcriptional difference alone does not demonstrate the agronomic superiority of PF-R-3. The comparison between PF-R-4 and PF-R-5 revealed a pronounced bias in differential regulation, with the number of up-regulated sequences far exceeding that of down-regulated sequences, indicating that PF-R-4 primarily drove gene activation relative to PF-R-5. Different PF treatments combinations exhibited clear divergence in the regulatory patterns and perturbation intensity of gene expression (Figure 1C).

### 3.3. GO and KEGG Enrichment Analysis of DEGs Under Different Treatments

**Figure 2:**
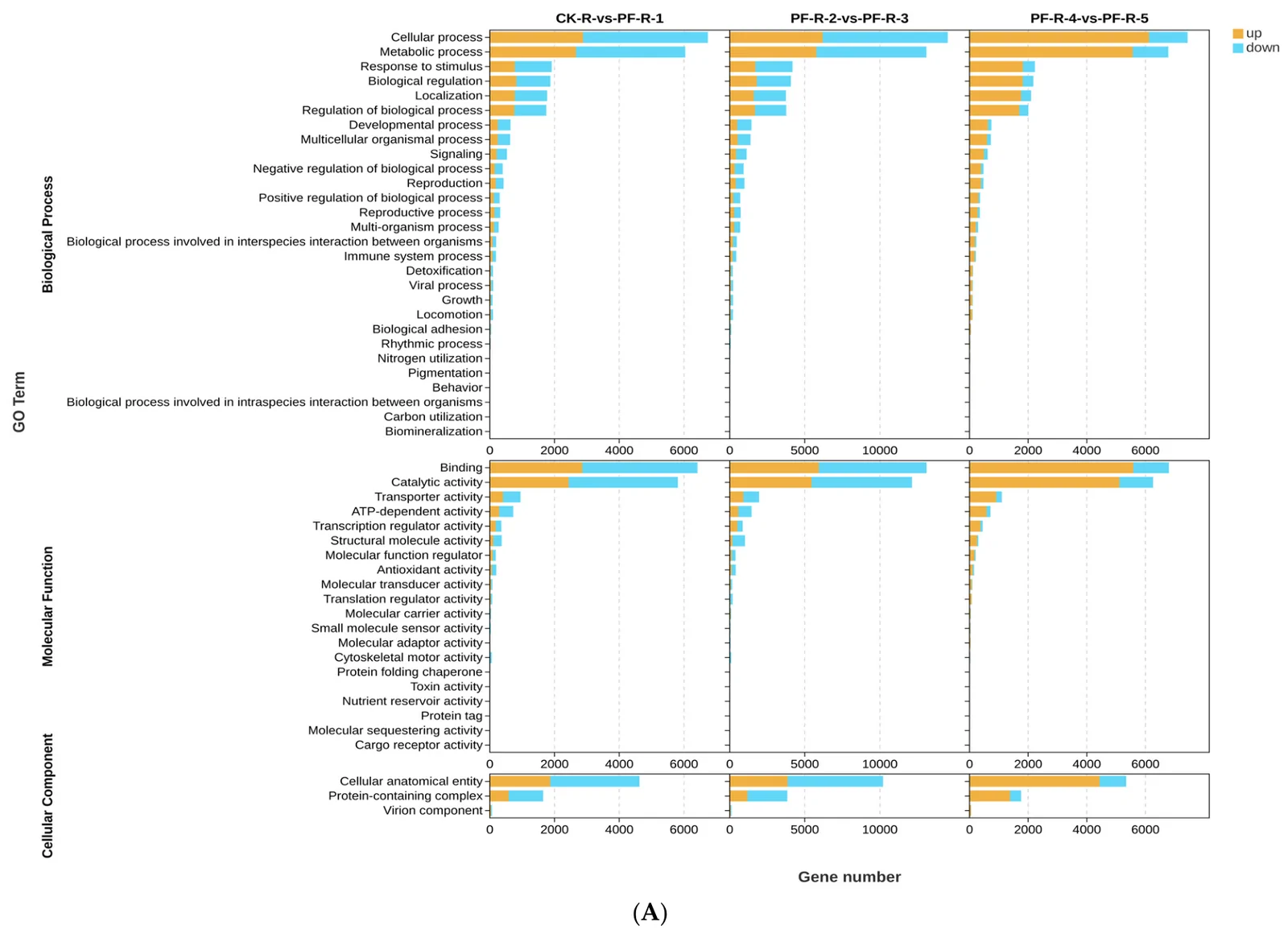

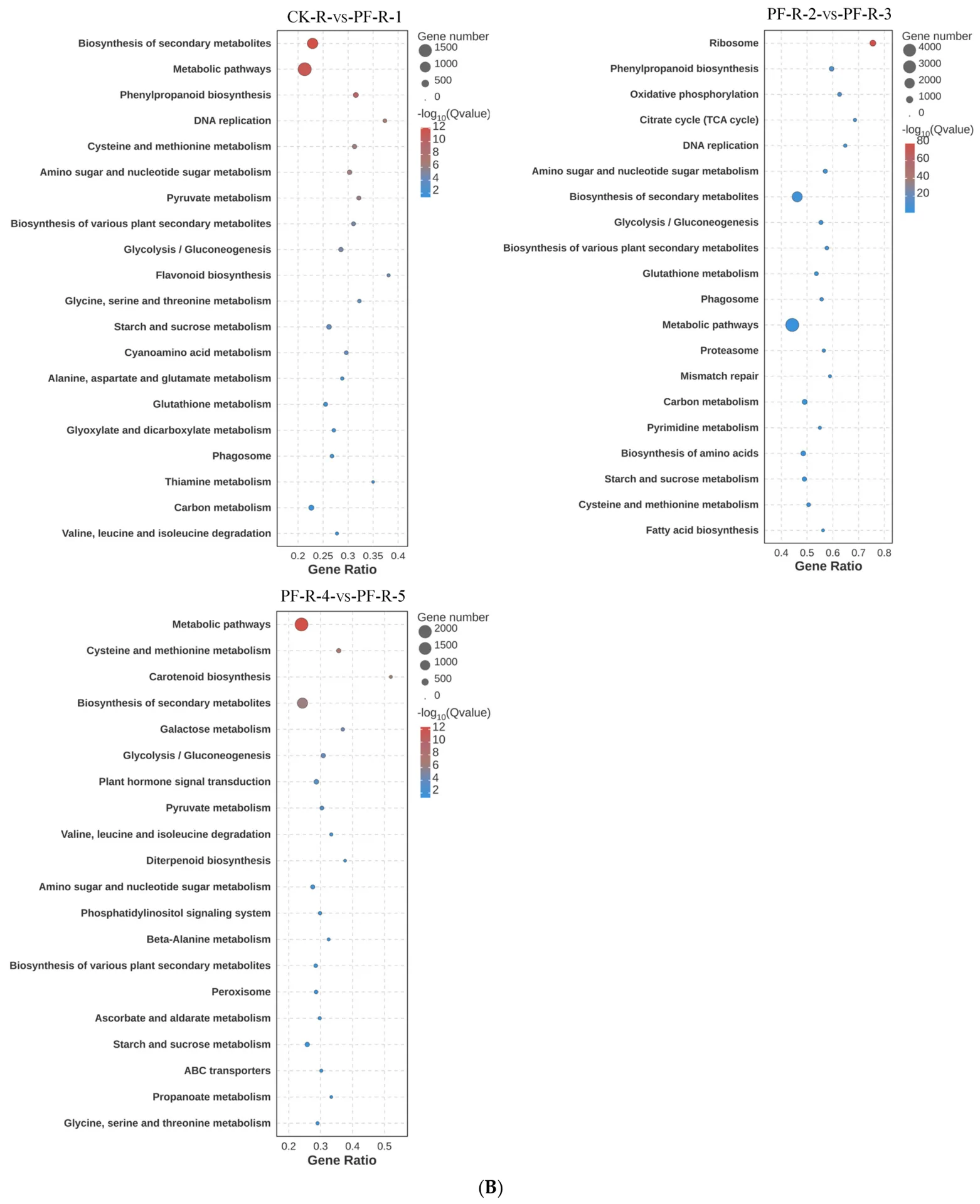
GO (**A**) and KEGG (**B**) enrichment analysis of DEGs under different treatments.

To investigate the core gene functions associated with different treatments during oat growth, GO and KEGG enrichment analyses were performed on the differentially expressed genes (DEGs). GO enrichment analysis results showed that in the comparison between CK and PF-R-1, the distribution of up- and down-regulated genes among core GO terms was balanced, indicating a bidirectional and equal regulation of basal metabolism-related functions by the treatment (Figure 2A). In the comparison between PF-R-2 and PF-R-3, the total number of DEGs annotated to GO terms was the highest, and down-regulated DEGs predominated in pathways related to cellular metabolism, catalytic activity, and cellular structure, which might be suggestive of a suppressed transcriptional program for these foundational biological processes under this treatment. In the comparison between PF-R-4 and PF-R-5, all core GO categories were predominantly represented by up-regulated genes; relative to PF-R-5, PF-R-4 significantly activated functional pathways associated with cellular metabolism, molecular catalysis, and cellular structure. The three treatment comparisons exhibited distinct regulatory patterns and perturbation intensities regarding fundamental biological functions. KEGG pathway enrichment analysis (Figure 2B) showed that the overall enrichment intensity of pathways in the comparison between CK and PF-R-1 was relatively low, and the differentially expressed genes were mainly enriched in pathways related to basic carbon and nitrogen metabolism, secondary metabolism, and DNA replication. In the comparison between PF-R-2 and PF-R-3, the pathway enrichment significance, gene enrichment ratio, and pathway coverage were the highest among the three comparisons. Pathways related to ribosomal protein translation, oxidative phosphorylation energy metabolism, and DNA damage repair were strongly and differentially enriched, indicating the most pronounced divergence in molecular metabolic networks between the two treatments. In the comparison between PF-R-4 and PF-R-5, in addition to basic metabolic pathways, pathways related to plant hormone signal transduction, carotenoid biosynthesis, and diterpenoid biosynthesis were specifically enriched. Secondary metabolic regulation was biased toward carotenoid and hormone signaling pathways, and no significant enrichment was observed in protein translation-related pathways.

### 3.4. Co-Expression Network Analysis of Differentially Expressed Genes

**Figure 3:**
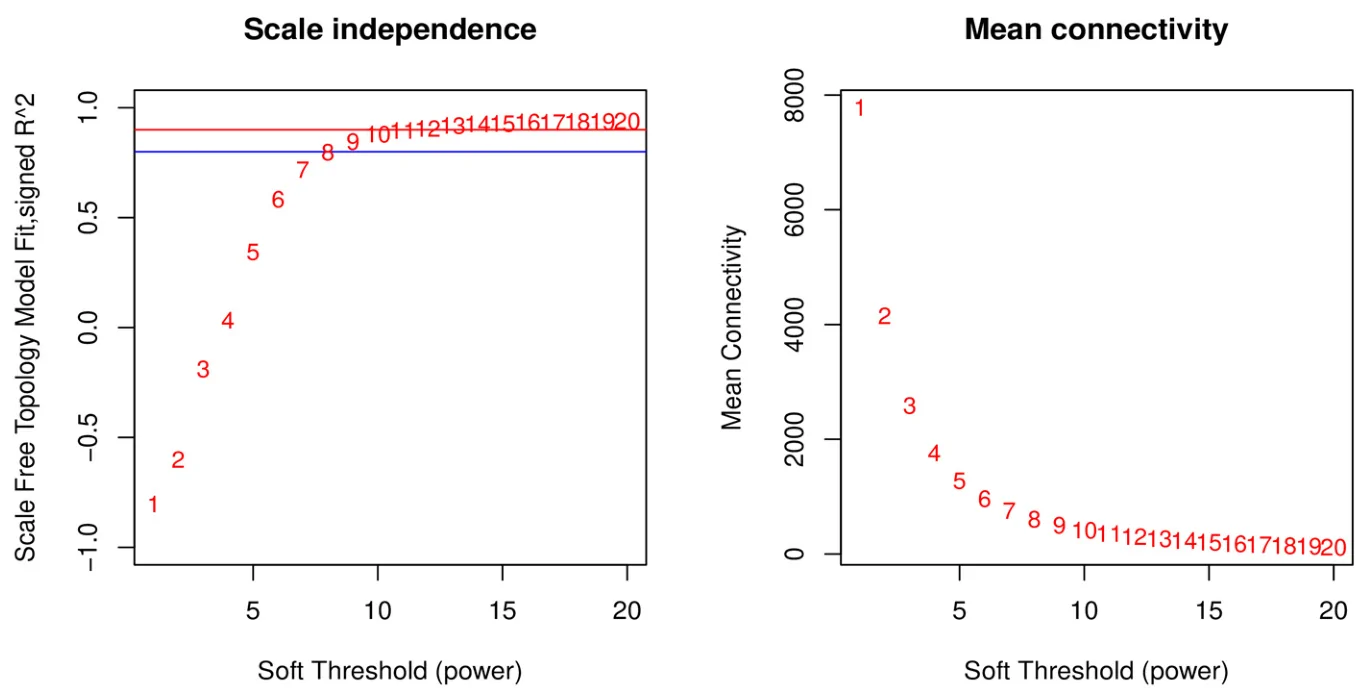
Determination of the soft-thresholding power (β).

Figure 3 presents the results of optimal soft-thresholding power selection in the WGCNA. The left panel shows that the scale-free topology fitting index (R^2^) increased continuously with increasing soft-thresholding power, and when power ≥ 9, indicating that the network satisfied the scale-free topology criterion.

Hierarchical clustering analysis of the weighted gene co-expression network (WGCNA) was performed based on the gene expression matrix. The gene clustering dendrogram showed that all genes were hierarchically clustered according to the similarity of their expression patterns, with the clustering height on the vertical axis representing the degree of gene expression dissimilarity. The majority of gene branches were merged within the height range of 0.4 to 0.8, suggesting the existence of stable and significant co-expression associations among genes and indicating good overall clustering quality of the data. Dynamic Tree Cut was applied for initial module detection, yielding a large number of finely partitioned small co-expression modules. To reduce module redundancy and simplify subsequent biological interpretation, the initial small modules were merged based on the correlation of module eigengenes (Merged dynamic), ultimately yielding several co-expression modules with pronounced size differences. Among them, the blue, black, and turquoise modules were the dominant modules with the highest gene counts, and the black module represented background genes without significant co-expression characteristics. The two module partitioning strategies were suitable for fine-scale sub-pathway mining and conventional downstream analyses such as module–trait association and functional enrichment, respectively, thereby providing a reliable grouping basis for the subsequent screening of key trait-associated genes and the elucidation of regulatory pathways.

**Figure 4:**
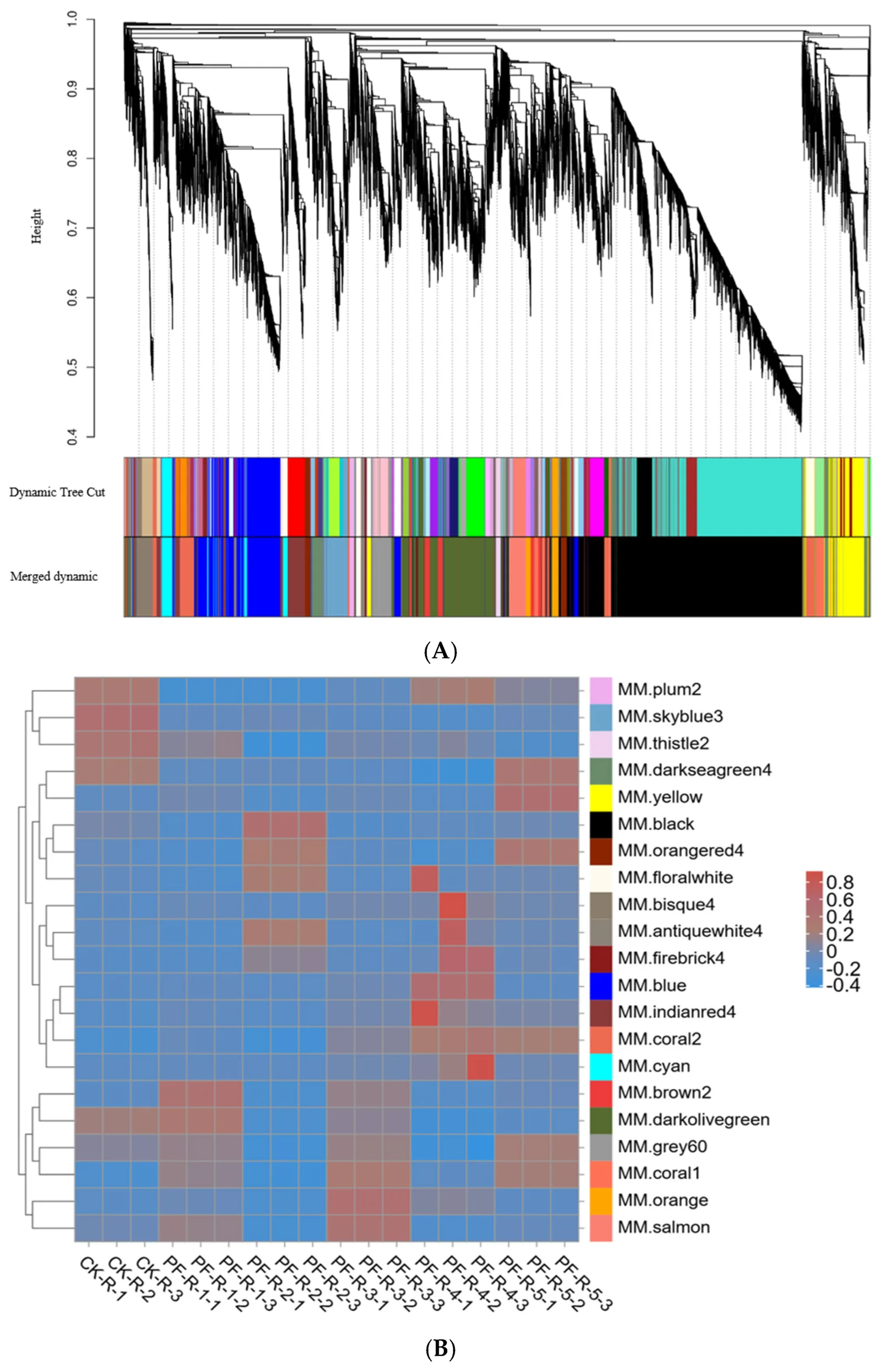
Systematic clustering tree and module division diagram (**A**); Correlation heatmap between module eigengenes and traits (**B**).

A module–module correlation heatmap was constructed based on module eigengenes (Figure 4B). The inter-module correlation coefficients showed pronounced differentiation. The MM.blue module exhibited significant negative correlations with modules such as MM.cyan, MM.indianred4, and MM.firebrick4, suggesting an antagonistic regulatory relationship in gene expression patterns between the two module groups, whereas the red-colored modules were highly positively correlated with one another, indicating that their genes coordinately participate in similar biological pathways. In conjunction with the module clustering dendrogram on the left, it was observed that modules with similar expression trends were clustered into the same branch, mutually corroborating with the correlation color block distribution pattern of the heatmap. This clearly revealed the synergistic and antagonistic regulatory relationships among the co-expression modules, providing a molecular association basis for the subsequent screening of interacting pathways and the identification of key functional modules. The coral2 module showed a significant positive correlation with the mycelial growth rate (r = 0.849, *p* = 8.45 × 10^−6^), and was therefore selected as the core regulatory module associated with fungal colonization.

### 3.5. GO and KEGG Enrichment Analysis of Root Module Genes

**Figure 5:**
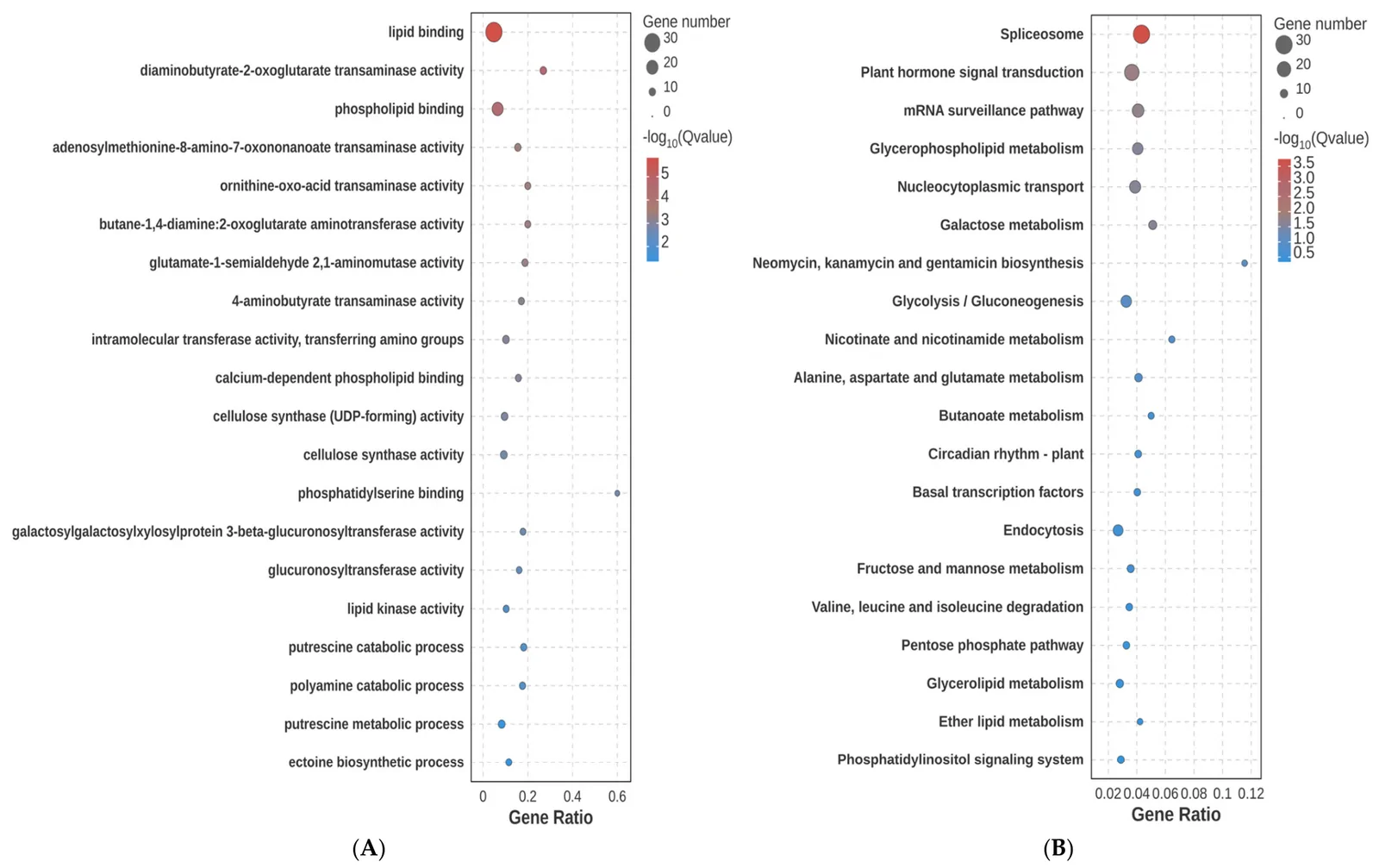
GO (**A**) and KEGG (**B**) enrichment analysis of genes in the Coral2 module.

**Figure 6:**
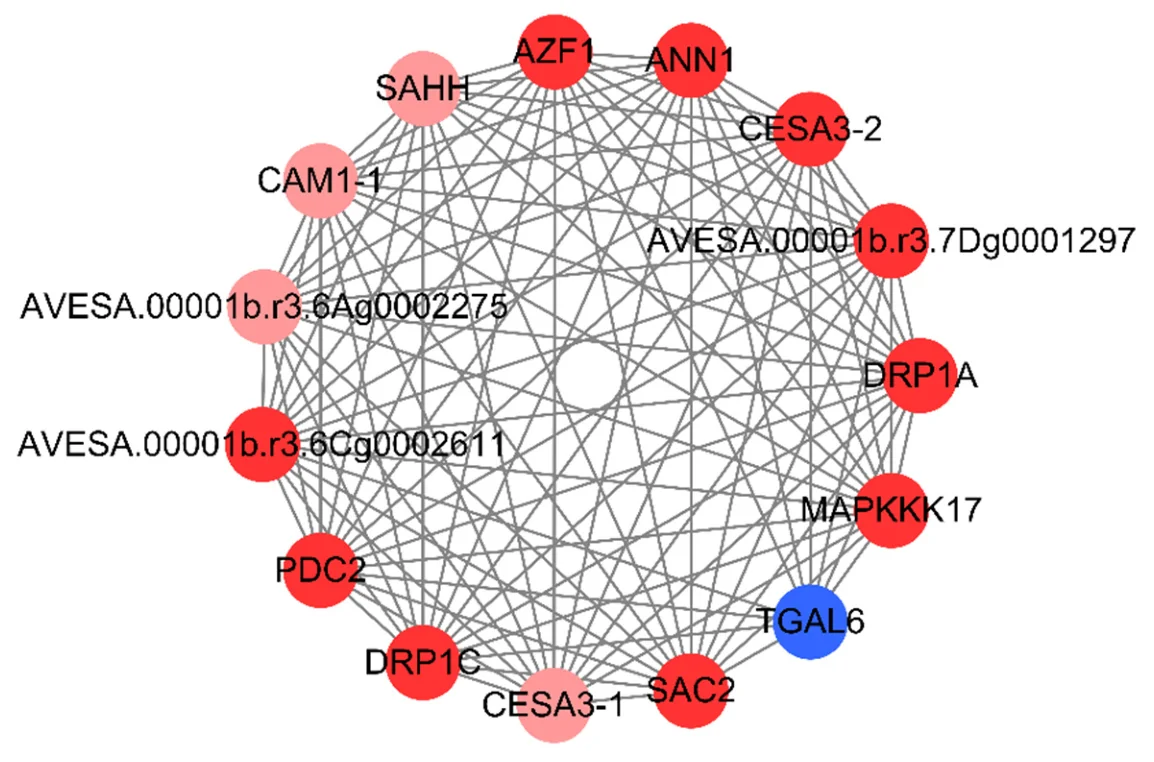
Interaction network of hub genes in the Coral2 module.

To elucidate the functions of the core root module (coral2), GO and KEGG enrichment analyses were performed. GO enrichment analysis results (Figure 5A) showed that, in response to different ratios of exogenous nutrient bag waste, the significantly enriched gene functions were mainly concentrated in three categories: amino acid metabolism and osmotic regulation, cell wall remodeling, and membrane lipid binding. Specifically, the enrichment of key enzyme genes such as diaminobutyrate transaminase, ornithine transaminase, and 4-aminobutyrate transaminase indicated that oat enhanced osmotic regulation and reactive oxygen species scavenging capacity by activating proline and GABA synthesis pathways. Meanwhile, the significant enrichment of genes related to putrescine catabolism further confirmed the active involvement of polyamine signaling in stress responses. Further KEGG enrichment analysis (Figure 5B) indicated that genes associated with RNA processing were enriched. Specifically, spliceosome, mRNA surveillance pathway, and nucleocytoplasmic transport pathway were among the top significantly enriched terms, suggesting that alternative splicing events played a crucial molecular switch role in the rapid adaptation of oat to complex soil environments, enabling the efficient production of multiple functional protein isoforms under energy-limited conditions. To screen core genes in the coral2 module (Figure 6), 12 algorithms of CytoHubba were used, and genes identified by more than half of the algorithms were selected as candidate genes. Ultimately, 15 hub genes were screened. Functional annotation results showed that CESA3-1 and CESA3-2 were annotated as cellulose synthases and are involved in cell wall synthesis; DRP1A and DRP1C are associated with vesicle trafficking and cell division; MAPKKK17 encodes a mitogen-activated protein kinase kinase kinase and participates in signal transduction; CAM1 is a calmodulin involved in calcium signaling; SAHH is related to the methionine cycle and methylation regulation; AZF1 is a zinc finger transcription factor, ANN1 is an annexin, SAC2 is a phosphoinositide phosphatase, and TGal6 may be involved in glycosylation modification. Collectively, the functions of these core genes span multiple levels, from signal transduction, cell wall synthesis, and metabolic regulation to transcriptional regulation, providing important candidate resources for studying the molecular mechanisms of oat.

### 3.6. WGA–PI Staining of Fungal Structures in Oat Roots

**Figure 7:**
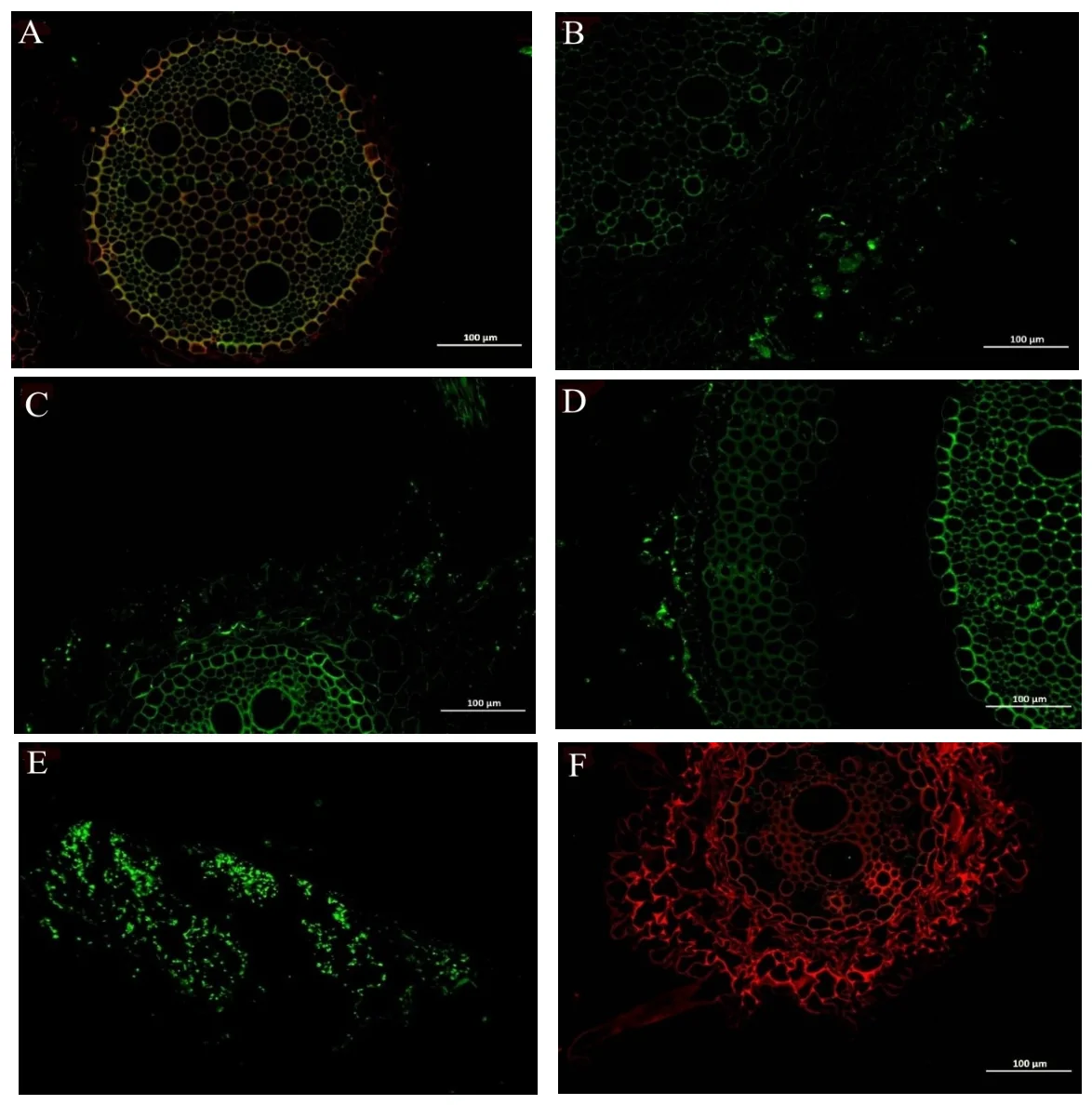
Staining status of fungal hyphae cells in different treatment groups. Note: (**A**) PF-R-1; (**B**) PF-R-2; (**C**) PF-R-3; (**D**) PF-R-4; (**E**) PF-R-5; and (**F**) CK (Uninoculated control) (Green fluorescent signals indicative of tubular fungal hyphae are observed in intercellular spaces and within root tissues). Scale bar = 100 µm.

The fluorescence microscopy results directly confirmed (Figure 7) that fungal hyphae could successfully colonize and invade oat root tissues. In the PF-R-1 treatment, which showed no observable fungal colonization in the root, the vascular and cortical structures remained intact, and no specific green fluorescence signal of exogenous fungi was detected. In the root sections of the inoculated treatment groups PF-R-2 to PF-R-5, green fluorescence signals characteristic of fungal hyphae were detected, with hyphae distributed in the intercellular spaces of the root cortex and within the tissues. Notably, in PF-R-4, intact hyphal rings embedded in the oat root tissue could be observed, while in PF-R-2, PF-R-3, and PF-R-5, fragmented or punctate fungal fluorescence signals were similarly distributed within the root cross-sections. These observations indicate that fungal hyphae were capable of penetrating the oat epidermis and colonizing the root interior, suggesting a close fungal association with oat roots.

## 4. Discussion

### 4.1. Differential Regulatory Effects of Exogenous Nutrient Bag Waste Ratios on Soil Fertility

This study demonstrated that returning ENB waste to the field significantly increased soil organic matter, nitrogen content, and microbial activity, which is largely consistent with previous findings on soil improvement through the incorporation of spent mushroom substrates. For instance, elevated soil salinity can adversely affect plant physiology by reducing water uptake, disrupting ion homeostasis and inducing oxidative stress, ultimately impairing crop growth and yield [21]. De Bonis et al. [22] found that the application of spent mushroom substrate (SPS) significantly enhanced soil organic matter, labile carbon, and enzyme activities, and promoted microbial activity. Tang et al. [23] reported in a paddy field study that the continuous application of different spent mushroom substrates for two years significantly increased the contents of soil organic carbon, total nitrogen, total phosphorus, and total potassium. Yang et al. [24] further confirmed that the addition of 1.5 kg m^−2^ of spent mushroom substrate increased soil total nitrogen by 21.2% and organic matter by 27.9%, and that the microbial nitrogen and phosphorus transformation efficiency was higher in the spent mushroom substrate treatment than in the control. In the present study, the PF-R-3 treatment (wheat + corncob + oat) consistently maintained medium to high levels of soil nutrients and biological activity (soil organic matter: 42.0–46.6 g kg^−1^) and exhibited the most gradual and balanced nutrient supply curve. This difference may be attributed to the C/N ratio and lignocellulose composition of the different organic material combinations.

### 4.2. Transcriptomic Responses of Oat Roots Reveal the Molecular Regulatory Advantages of PF-R-3 Treatment

Transcriptomic analysis provided molecular-level evidence for understanding the differential effects among treatments. The enrichment of genes in the oxidative phosphorylation pathway may suggest a potential shift toward enhanced mitochondrial energy metabolism in roots, which could potentially support the active transport of nutrients across root cell membranes. However, direct measurements of mitochondrial respiration, ATP content, or transporter activity were not performed in this study, and this interpretation remains tentative. Adu et al. [25] found in a transcriptomic study on rice responses to low nitrogen that plants adjust their root system architecture and transcriptome composition according to nitrogen status, including the expression of genes related to nitrogen uptake and assimilation. Compared with CK, the PF-R-3 treatment significantly upregulated NRT and AMT family genes in the roots (*p* < 0.05), indicating a stronger nitrogen acquisition capacity. This is consistent with the significantly higher soil alkali-hydrolyzable nitrogen content under PF-R-3 (211.4–231.7 mg kg^−1^) than under CK (*p* < 0.05). Through the upregulation of transcriptional responses consistent with potential enhancement of N transport, the roots enhanced their capacity to actively absorb nitrate and ammonium nitrogen from the soil, thereby providing sufficient nitrogen sources for protein synthesis in the aboveground parts.

WGCNA further identified the coral2 core module, whose genes were predominantly enriched in functions such as transmembrane transport, transporter activity, and plant hormone signal transduction, which were highly consistent with the physiological roles of root nutrient uptake and ion homeostasis maintenance. The identification of this module provides targets for future exploration of key candidate genes underlying oat root responses under waste return conditions. In a study on rice responses to low nitrogen, WGCNA classified 25,377 genes into 14 modules, two of which were significantly associated with nitrogen uptake and utilization [25]. In a study on poplar nitrate stress responses, WGCNA revealed the critical role of the ERF2B–NRT2.1C module as a core regulatory factor in nitrate uptake and assimilation [26]. We have expanded the WGCNA-related discussion by including additional examples from maize [27] and poplar [28], and by comparing the functional characteristics of our coral2 module with nutrient-responsive modules reported in these systems. The coral2 module identified in this study was functionally similar to core nutrient-responsive modules previously reported in rice and poplar, suggesting that the molecular responses of plants to the return of different types of organic materials may involve conserved regulatory network.

However, it should be noted that root samples were collected from mixed stands of three oat cultivars and pooled before RNA extraction; therefore, the observed transcriptional responses cannot be attributed to any single cultivar and should be interpreted as the integrated response of the mixed stand.

### 4.3. Ecological Implications of Morchella Mycelium Application

Fungal mycelium is rich in structural components such as chitin and β-glucan. Its incorporation into soil has been reported to alter microbial community composition and soil functionality. For instance, ENB waste application improved vineyard soil multifunctionality and modified bacterial and fungal community structures [29], while pear sawdust ENB was associated with reduced relative abundance of soilborne fungal genera such as *Fusarium* and *Aspergillus*, alongside increased abundance of bacteria including *Pedobacter*, *Pseudomonas*, and *Devosia* in orchard soils [30].

In the present study, the enrichment of plant–pathogen interaction pathway-related genes in oat roots colonized by Morchella mycelium suggests a potential molecular dialogue between the fungus and the host plant. As a saprophytic fungus, Morchella is not a typical plant pathogen; thus, its presence within root tissues might be perceived by the plant as a non-threatening microbial association. Whether such recognition leads to defense priming or growth modulation, however, remains to be experimentally validated.

## 5. Conclusions

This study investigated the effects of different ENB waste-based substrate combinations on soil properties and root transcriptional responses in oat cultivation. The incorporation of ENB waste into field soil was associated with increased organic matter, nitrogen content, and microbial activity, along with improvements in soil saline–alkali conditions. Among the treatments, PF-R-3 (wheat + corncob + oat) displayed a relatively stable nutrient supply profile during the growing season, with most soil parameters maintained at moderate-to-high levels. Transcriptomic comparisons revealed that the PF-R-3 vs. PF-R-2 contrast yielded the highest number of differentially expressed genes, with enrichments in pathways related to ribosomal translation, oxidative phosphorylation, and DNA repair. These transcriptional patterns suggest a potential link between this substrate combination and enhanced basal metabolism and energy utilization at the gene expression level. In addition, the coral2 WGCNA module, enriched in transmembrane transport and phytohormone signaling genes, may contribute to root nutrient uptake and stress adaptation. Fluorescence microscopy confirmed the presence of Morchella mycelium in association with oat roots, raising the possibility of fungal–root interactions in these regulatory processes. Taken together, these findings provide molecular-level clues for selecting ENB-based substrates in oat production. However, because grain yield and quality data are not yet available, and the functional roles of root-associated Morchella remain to be characterized, PF-R-3 should be considered a promising candidate rather than a finalized recommendation. Further field trials integrating agronomic performance and long-term soil health assessments are required before practical application can be advised.

## Figures and Tables

**Table 1:** Different processing and addition levels of exogenous nutrient bag waste.

Treatment	Wheat (%)	Corn Cob (%)	Oat Grass (%)	Alfalfa (%)
PF-R-1	50	40	-	-
PF-R-2	30	30	-	30
PF-R-3	30	30	30	-
PF-R-4	30	-	-	60
PF-R-5	30	-	60	-

**Table 2:** Soil physicochemical properties.

Time	Treatment	SOM (g/kg)	TN (g/kg)	AN (mg/kg)	pH	EC (μs/cm)
Before sowing	PF-R-1	46.2 ± 0.01 a	2.0 ± 0.01 a	220.7 ± 0.2 a	8.1 ± 0.02 b	1115.5 ± 20.5 c
	PF-R-2	43.2 ± 0.5 ab	2.0 ± 0 b	216.3 ± 1.0 a	8.1 ± 0.01 b	2390.0 ± 42.4 a
	PF-R-3	41.9 ± 0.2 b	2.0 ± 0 b	211.4 ± 0 a	8.0 ± 0.01 c	938.5 ± 6.4 d
	PF-R-4	41.1 ± 0.3 b	1.9 ± 0.01 c	195.3 ± 2.5 b	8.1 ± 0.01 c	326.0 ± 21.2 e
	PF-R-5	32.5 ± 0.6 c	1.5 ± 0.01 d	159.1 ± 4.7 c	8.1 ± 0.01 b	207.5 ± 0.7 f
	CK	44.9 ± 1.0 a	2.2 ± 0.03 a	195.3 ± 2.5 b	8.3 ± 0.05 a	1284.0 ± 19.8 b
81 d	PF-R-1	47.2 ± 0.3 b	2.3 ± 0.04 a	241.3 ± 6.2 a	8.3 ± 0 b	298.5 ± 3.5 a
	PF-R-2	47.7 ± 0.1 b	2.2 ± 0 a	219.6 ± 2.7 bc	8.1 ± 0.04 c	222.5 ± 10.6
	PF-R-3	46.6 ± 0.2 b	2.1 ± 0.03 b	231.7 ± 1.6 ab	8.4 ± 0.01 a	292.0 ± 8.5 a
	PF-R-4	51.0 ± 0.5 a	2.3 ± 0.04 a	244.1 ± 4.2 a	8.0 ± 0.01 c	274.5 ± 5.0 b
	PF-R-5	41.2 ± 0.2 d	1.7 ± 0.01 c	180.6 ± 0.5 d	8.0 ± 0.05 c	165.8 ± 3.7 d
	CK	44.5 ± 0.5 c	2.3 ± 0.02 a	209.6 ± 2.0 c	8.5 ± 0.01 a	225.5 ± 0.7 c
159 d	PF-R-1	37.4 ± 0.3 d	2.2 ± 0.03 d	151.2 ± 2.0 e	8.6 ± 0.08 a	262.0 ± 2.8 c
	PF-R-2	45.1 ± 0.1 b	2.5 ± 0.03 b	196.0 ± 1.5 b	8.1 ± 0.01 d	276.0 ± 5.7 b
	PF-R-3	43.9 ± 0.2 bc	2.4 ± 0.06 bc	178.3 ± 2.2 c	8.3 ± 0.01 bc	282.0 ± 5.7 ab
	PF-R-4	43.2 ± 0.5 bc	2.4 ± 0.01 c	162.9 ± 2.2 d	8.3 ± 0.08 bc	288.0 ± 5.7 a
	PF-R-5	48.8 ± 0.5 a	2.8 ± 0.05 a	212.1 ± 1.5 a	8.2 ± 0.01 c	281.0 ± 1.4 ab
	CK	41.9 ± 0.08 c	2.4 ± 0.01 bc	164.7 ± 4.2 d	8.4 ± 0.01 b	256.0 ± 1.4 c

Note: Values are mean ± SD (*n* = 3). Different lowercase letters in the same column denote significant differences among treatments within the same sampling time (Tukey’s HSD. test, *p* < 0.05).

**Table 3:** Soil biological properties.

Time	Treatment	UR (μg/g/h)	PRO (μg/g/h)	CAT (ml/g/h)	MBC (mg/kg)	MBN (mg/kg)	MBP (mg/kg)
Before sowing	PF-R-1	296.2 ± 5.4 a	0.25 ± 0.01 a	0.8 ± 0.1 e	751.1 ± 12.6 a	42.6 ± 2.0 a	25.2 ± 0.5 a
	PF-R-2	229.4 ± 0.4 b	0.21 ± 0 b	2.8 ± 0.09 c	686.6 ± 0.7 b	38.3 ± 0.3 b	20.1 ± 0.8 b
	PF-R-3	215.8 ± 4.2 bc	0.18 ± 0 c	1.6 ± 0.27 d	666.5 ± 10.2 b	33.4 ± 2.9 c	18.3 ± 0.8 c
	PF-R-4	202.6 ± 9.5 c	0.15 ± 0 d	4.5 ± 0.03 b	643.0 ± 4.1 b	32.1 ± 0.7 c	17.3 ± 0.02 c
	PF-R-5	111.9 ± 8.2 d	0.13 ± 0 e	5.3 ± 0.01 a	456.8 ± 13.7 d	25.0 ± 0.2 d	11.0 ± 0.35 d
	CK	215.3 ± 10.3 bc	0.21 ± 0 b	2.6 ± 0.04 c	515.5 ± 10.1 c	30.7 ± 0.2 c	21.0 ± 0.72 b
81 d	PF-R-1	253.3 ± 2.5 b	0.2 ± 0.01 a	4.1 ± 0.02 e	729.3 ± 19.2 b	55.0 ± 3.4 b	29.2 ± 0.2 c
	PF-R-2	236.9 ± 0.2 c	0.14 ± 0 d	4.4 ± 0.01 c	632.9 ± 27.6 c	43.3 ± 4.5 c	22.7 ± 0.9 d
	PF-R-3	255.8 ± 0.22 b	0.17 ± 0 c	4.7 ± 0.02 c	695.1 ± 1.6 b	44.8 ± 0.69 c	29.5 ± 1.5 c
	PF-R-4	279.8 ± 6.5 a	0.2 ± 0 b	4.6 ± 0.04 d	958.0 ± 30.5 a	66.3 ± 2.2 a	33.7 ± 0.5 a
	PF-R-5	127.8 ± 3.4 e	0.17 ± 0 c	5.7 ± 0.06 a	611.7 ± 1.8 c	44.1 ± 1.0 c	22.1 ± 1.6 d
	CK	219.1 ± 4.2 d	0.19 ± 0.01 b	5.3 ± 0.06 b	706.3 ± 27.1 b	56.3 ± 1.0 b	31.7 ± 0.3 b
159 d	PF-R-1	189.9 ± 2.5 b	0.21 ± 0 c	5.8 ± 0.05 b	215.1 ± 4.1 d	15.2 ± 0.1 c	15.4 ± 0.5 b
	PF-R-2	216.5 ± 2.5 a	0.23 ± 0 b	5.7 ± 0 c	382.0 ± 4.0 b	37.2 ± 1.5 a	18.1 ± 0.09 a
	PF-R-3	187.2 ± 1.7 b	0.2 ± 0.01 c	5.8 ± 0.09 b	355.4 ± 5.4 c	33.3 ± 1.3 b	13.5 ± 0 c
	PF-R-4	179.0 ± 0.3 c	0.23 ± 0 b	6.1 ± 0.04 a	346.1 ± 1.7 c	32.1 ± 0.9 b	13.5 ± 0.08 c
	PF-R-5	212.3 ± 3.9 a	0.25 ± 0 a	5.8 ± 0.07 b	449.6 ± 1.5 a	39.5 ± 1.6 a	16.9 ± 0.9 ab
	CK	177.6 ± 2.5 c	0.20 ± 0 c	6.0 ± 0.03 a	189.0 ± 10.5 e	15.3 ± 1.0 c	10.5 ± 0.62 d

Note: Values are mean ± SD (*n* = 3). Different lowercase letters in the same column denote significant differences among treatments within the same sampling time (Tukey’s HSD. test, *p* < 0.05).

## Data Availability

The original contributions presented in this study are included in the article. Further inquiries can be directed to the corresponding author.
